# Multifunctional Self-Pumping Janus Dressing for Exudate Management and Diabetic Wound Healing

**DOI:** 10.3390/biom16060902

**Published:** 2026-06-18

**Authors:** Yingnan Yue, Naoyuki Chado, Rike Rachmayati, Rie Wakabayashi, Noriho Kamiya, Shinichi Aishima, Hiroyuki Ijima, Yasuhiro Ikegami

**Affiliations:** 1 Department of Chemical Engineering, Faculty of Engineering, Graduate School, Kyushu University, 744 Motooka, Nishi-ku, Fukuoka 819-0395, Japan; yue.yingnan.308@s.kyushu-u.ac.jp (Y.Y.); naoyukichadou@gmail.com (N.C.); ijima@chem-eng.kyushu-u.ac.jp (H.I.); 2 Department of Applied Chemistry, Graduate School of Engineering, Kyushu University, 744 Motooka, Nishi-ku, Fukuoka 819-0395, Japan; rachmayati.rike.524@s.kyushu-u.ac.jp (R.R.); wakabayashi.rie.122@m.kyushu-u.ac.jp (R.W.); kamiya.noriho.367@m.kyushu-u.ac.jp (N.K.); 3 Division of Biotechnology, Center for Future Chemistry, Kyushu University, 744 Motooka, Nishi-ku, Fukuoka 819-0395, Japan; 4 Department of Scientific Pathology, Graduate School of Medical Sciences, Kyushu University, 3-1-1 Maidashi, Higashi-ku, Fukuoka 812-8582, Japan; aishima.shinichi.476@m.kyushu-u.ac.jp

**Keywords:** multi-functional nanofibrous dressing, Janus nanofiber, bioactive agent release, diabetic wound healing

## Abstract

Diabetic chronic wounds are often accompanied by excessive wound exudate maceration, which prolongs the inflammatory phase and increases the risk of infection. Such a complex wound microenvironment imposes more stringent requirements on multifunctional wound dressings. A multifunctional Cur Janus nanofibrous dressing is developed by integrating an electrospun poly(ε-caprolactone)/gelatin hydrophilic layer with a curcumin (Cur)-loaded PCL hydrophobic layer. Janus structure with asymmetric wettability, which exhibited unidirectional liquid transport properties both in vitro and in vivo. Its unique structure also makes it possible to carry both hydrophilic and hydrophobic drugs at the same time. The incorporation of curcumin endows the dressing with antibacterial and antioxidant functionalities, offering the potential to modulate the inflammatory microenvironment of diabetic chronic wounds. Furthermore, the wound healing ability and anti-inflammatory effects of Cur Janus nanofibers were evaluated in a diabetic mouse model. The results showed that Cur Janus nanofibers significantly reduced wound area, increased the proportion of pro-healing M2 macrophages, shortened the inflammatory phase, and ultimately accelerated diabetic wound healing. This work provides a multifunctional and scalable platform for advanced wound dressing design. Its excellent antibacterial, antioxidant (ROS scavenging) and anti-inflammatory (macrophage phenotype M1 to M2) properties, combined with the unidirectional fluid transport and dual-release potential of hydrophilic and hydrophobic drugs, demonstrate broad prospects in the management of diabetic wounds.

## 1. Introduction

The normal wound healing process is a succession and overlap of several multidimensional phases of hemostasis, inflammation, proliferation, and remodeling [1]. Wound exudate is the fluid produced by wounds once hemostasis has been achieved, which contains electrolytes, nutrients, proteins, inflammatory mediators, proteinase, growth factors and waste products [2,3]. Exudate is an essential component of the wound healing, which can not only remove dead tissues and promote new tissue growth, but also exudate provides the moisture for ‘moist wound healing’ [4]. However, sometimes, exudate can also be problematic. Peri-wound maceration is defined as the softening and breaking down of epidermis resulting from prolonged exposure to wound exudate that contains proteases, and can occur in any exudating chronic wound [5]. Excessive exudate especially in diabetic chronic wound can saturate the wound bed and cause maceration, which will lead to wound degeneration, infection, and persistent inflammatory responses [6,7]. Infection is a common complication in damaged skin, and preventing it is critical, as bacteria and their products (e.g., endotoxins, metalloproteinases) can disrupt all stages of wound healing [8,9]. During the inflammatory phase of wound healing, ROS (reactive oxygen species) will be produced in the process of the immune system (macrophages, neutrophils) fighting pathogens. However, excessive ROS can increase oxidative stress, prolong inflammation and delay wound healing. Therefore, good wound management relies on the control of exudate and reduction in infection and oxidative stress [10,11,12].

Traditional dressing is a vital part with the ability to manage the excessive biofluid, which is usually prepared from hydrophilic material, such as cotton, cellulose and hydrogel [13]. However, traditional hydrophilic dressing inevitably leaves biofluid at the interface between wound and dressing. Notably, biofluid remaining in the wetted hydrophilic traditional dressing could penetrate in reverse when contact with the wound for a long time. This will lead to the overhydration of wound and delay wound healing [14]. To overcome this predicament, Janus materials with asymmetric wettability and their resulting unidirectional liquid transport ability have recently attracted increasing attention [15,16,17]. Previously, because of their unidirectional biofluid transport ability, they also provide an idea for diabetic wound dressing, which enable the management of excessive exudate and prevent diabetic wound rewetting [18,19,20]. Among them, electrospinning is a powerful method for constructing the Janus structure which is formed by organizing the deposition of nanofibers on a collector [21,22,23]. Electrospun nanofibers have been widely applied in wound dressings due to their high porosity, large specific surface area, and capacity to incorporate various bioactive agents [24,25]. However, these Janus nanofibers wound dressing rarely consider the drug loading properties and their insufficient multifunctional properties also limited their value in diabetic wound, such as antibacterial, antioxidant and anti-inflammatory properties. Hydrophilic drugs and bioactive molecules were used for wound healing for their efficient drug release and therapeutic effect in an aqueous environment, which is beneficial for therapy during wound healing [26,27,28]. However, long-term treatment with drugs and bioactive molecules is pivotal for diabetic chronic wound with a complex wound microenvironment. Curcumin, a naturally occurring polyphenolic compound extracted from the rhizome of Curcuma longa, is a bioactive hydrophobic molecule with well-documented antioxidant and antibacterial properties [29,30,31,32]. Poly(ε-caprolactone) (PCL) is a hydrophobic polymer which is largely applied in biomaterial, as well as biomedical applications. Its slow degradation characteristics in water make it an ideal material for curcumin sustain release. It has been reported that curcumin was loaded into PCL nanofibers for wound-healing applications, which exhibited the sustained release of curcumin to the wound site and improved wound reduction [33,34].

**Figure 1:**
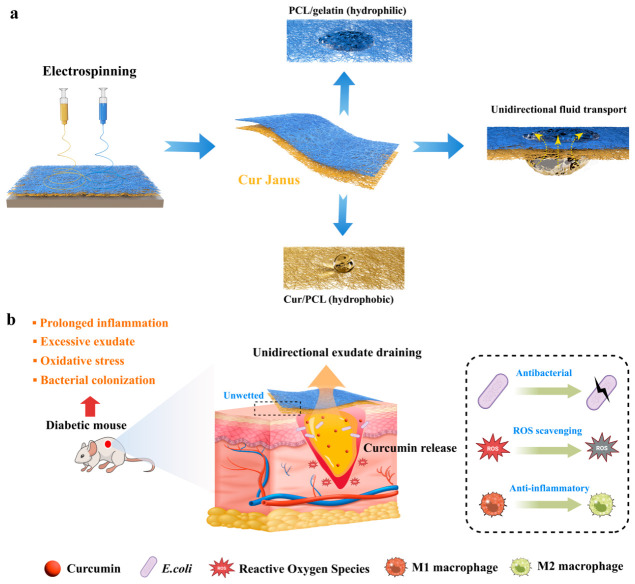
Schematic illustration of Cur Janus nanofiber and its application for diabetic wound healing. (**a**) Janus wound dressing based on PCL/gelatin–curcumin/PCL nanofiber and its unidirectional fluid transportation property. (**b**) Schematic illustration of diabetic wound treated with Cur Janus nanofiber.

Herein, we developed a multifunctional Janus nanofibrous dressing with unidirectional biofluid transport ability, dual release of hydrophilic and hydrophobic drugs, antibacterial, antioxidant and effective anti-inflammatory properties. This system is composed of an electrospun poly(ε-caprolactone)/gelatin hydrophilic layer and an electrospun curcumin-loaded PCL hydrophobic layer, which could realize effectively biofluid unidirectional transportation and provide a platform for the dual delivery of hydrophilic and hydrophobic drugs (Figure 1a). The Janus structure enables the unidirectional removal of excessive wound exudate, thereby preventing maceration of the wound site. In addition, the incorporation of curcumin into the hydrophobic layer prolongs its retention at the wound site without compromising the functionality of the Janus structure. Furthermore, a full-thickness diabetic wound model in mice was employed to evaluate the wound healing efficiency and inflammatory response following treatment with the Cur Janus nanofibrous dressing (Figure 1b). This study provides a multifunctional and scalable platform for versatile wound dressing design and offers new insights into the application of Janus architectures for diabetic wound management.

## 2. Materials and Methods

### 2.1. Materials

The PCL (Mn, 80 kDa) and gelatin (type 1A) were purchased from Sigma Aldrich (St. Louis, MO, USA). Hexafluoroisopropanol (HFIP) were from FUJIFILM Wako Pure Chemical Corporation, Osaka, Japan. Isoflurane (099-06571) were purchased from Fujifilm Wako Chemicals Japan Corporation (Fukuoka, Japan). NIH/3T3 clone 5611 (JCRB0615) was purchased from JCRB cell bank (Osaka, Japan). Cell counting kit-8 (AJ006) was purchased from Dojindo Laboratories (Kumamoto, Japan). EDC (Peptide Institute Inc., Osaka, Japan). The following were purchased: NHS (FUJIFILM Wako Pure Chemical Corporation, Osaka, Japan); fluorescence sodium (Novartis pharmaceuticals corporation, Tokyo, Japan); cyanoacrylate instant glue (Taoka chemical Co., Ltd., Osaka, Japan). CD-68 (ab125212), and CD 206 (ab234000) antibodies were purchased from Abcam (Cambridge, UK). Streptozotocin (Cat. No. [S1312], Selleckchem, Houston, TX, USA).

The following materials were acquired: water purification system (Direct-Q 3UV, Merck, Darmstadt, Germany); freezer dryer (WYELA FDU-1200, Tokyo Rikakikai, Tokyo, Japan); Benchtop scanning electron microscope (JCM-7000, JEOL Ltd., Tokyo, Japan); 8 mm skin biopsy puncture (Kai Industries, Tokyo, Japan); small animal anesthetizer (TK-36, Bio machinery, Seki-shi, Japan); inverted light microscope (CKX53, Olympus, Tokyo, Japan); and tensile and compression test machine (LTS-50N-S100, Minebeamitsumi, Tokyo, Japan).

### 2.2. Methods

#### 2.2.1. Janus and Cur Janus Nanofiber Preparation and Characterization

First, 5 wt% PCL/gelatin (1:2 *w*/*w*) dissolved in HFIP and electrospun on 5.5 cm × 5.5 cm square aluminum plate (voltage: 15 kV; needle: 18G; needle-to-collector distance: 12 cm; flow rate: 2 mL/h) for 1 h to fabricate PCL/gelatin nanofiber sheet through a custom-built electrospinning set up (syringe pump 11, Harvard apparatus, Holliston, MA, USA). The PCL/gelatin nanofiber sheet was punched into ∅15 mm round pieces. Nanofiber was put into 24-well plate, then covered with 400 μL of EDC/NHS ethanol solution (EDC: 10 mg/mL, NHS: 6 mg/mL) at 4 °C in dark environment overnight. The crosslinked nanofiber was washed with 500 μL of EtOH twice, subsequently replaced by tert-butanol. Nanofibers ware put into −80 °C freezer for 2 h and freeze dried for 8 h to get PCL/gelatin nanofiber (hydrophilic layer). PCL solution (Mass fraction 10 wt%) was prepared by dissolving PCL into chloroform/DMF (9:1, *v*/*v*) mixture and electrospun on PCL/gelatin nanofiber sticked onto rotating collector (needle-to-collector distance: 15 cm; collector diameter: 75 mm, rotating speed: 40 rpm) for 0, 2.5, 5 and 7.5 min to fabricate Janus nanofiber (Janus-2.5, Janus-5 and Janus-7.5), respectively.

Curcumin (10 wt% of PCL) and PCL were dissolved in chloroform/DMF (9:1, *v*/*v*) to get curcumin/PCL solution (total mass fraction 10 wt%, 9.09 wt% PCL, 0.91 wt% curcumin) and electrospun on 5.5 cm × 5.5 cm square aluminum plate or PCL/gelatin nanofiber sticked at rotating collector (needle-to-collector distance: 15 cm; collector diameter: 75 mm, rotating speed: 40 rpm) for 5 min to get curcumin-containing PCL (Cur PCL) or Janus (Cur Janus) nanofiber. Nanofibers were vacuum dried for 24 h to evaporate the solvent completely and were sputter-coated with platinum, and observed by SEM.

To determine the water vapor permeability of the nanofibers (PCL/gelatin, PCL, Cur Janus), the nanofibers were cut into round pieces (∅12 mm). Five hundred microliter of deionized water was added to a 0.5 mL tube, and the nanofiber was placed over the tube opening. The nanofiber was then sealed and secured to the tube using a custom-designed assembly consisting of a syringe component, silicone sealant, and clips. The assembled devices were incubated at 32 °C for 24 h. The water vapor transmission rate (WVTR) was calculated using the following equation. All measurements were performed in triplicate (*n* = 3).

(1) $$\mathrm{WVTR}\;(g/m^{2}/\mathrm{day})=\frac{W_{0}-W_{t}}{test\;area\times time}$$

W_0_ is the initial weight, W*_t_* is the weight after 24 h.

The mechanical properties of the nanofibers were tested using a tensile- and compression-testing machine. The sample total size was 30 mm × 10 mm with the test section being 20 mm in length, 5 mm in width, and 40 μm in thickness. Tensile strength–strain curve was plotted at a tensile speed of 10 mm/min. The maximum value indicates the maximum tensile strength of nanofiber.

#### 2.2.2. Unidirectional Transportation Behavior Observation

Five microliter of methylene blue aqueous solution (0.01 wt%) and 5 µL of pure water was dropped on the hydrophobic surface of Janus nanofiber (PCL side); then, contact angle and transport speed through nanofiber were recorded. Similarly, 5 μL of fluorescence sodium PBS solution (1 wt% fluorescence sodium) was contact to PCL/gelatin nanofiber, hydrophilic side of Janus nanofiber (PCL/gelatin side) and hydrophobic side of Janus nanofiber (PCL side) for observing the unidirectional transportation. 395 nm UV light was used for exciting the green fluorescence.

Wounds (∅8 mm) were punched on the back of mouse, then added with 50 μL of fluorescein sodium PBS solution (0.05 wt%). The wounds were covered with Janus nanofiber, PCL nanofiber and Gauze (∅12 mm) for 10 min followed by observation under fluorescence microscope.

#### 2.2.3. In Vitro Release of Curcumin from Cur PCL Nanofiber

Three milligram of Cur PCL nanofiber was immersed into 10 mL of 0.5 vol% Tween 80-containing PBS solution (releasing media) at 32 °C. The absorbance (λ: 427 nm) of the releasing media were measured at different time point (0.5, 1, 2, 4, 6, 24, 48 and 96 h). Released curcumin weight was calculated in releasing media was calculated by the calibration curve with known concentration, absorbance and volume (10 mL). Cumulative release of curcumin is calculated by the formula below:

(2) $$\mathrm{Cumulative}\;\mathrm{release}\;(\%)\;=\;\frac{Weight\;of\;released\;Curcumin}{Theoretical\;amount\;of\;Curcumin}\times 100$$

#### 2.2.4. In Vitro Release of Hydrophilic Drug from Janus Nanofiber

Fluorescein sodium was added to the 5 wt% PCL/gelatin solution to a final concentration of 0.4 mg/mL was mixed overnight. The mixture was electrospun on 5.5 cm × 5.5 cm plate collector (needle: 18 G, flow rate: 2 mL/h, distance: 12 cm, voltage: 15 kV) for 1 h to fabricate PCL/gelatin nanofiber. PCL/gelatin nanofiber punched into 12 mm in diameter was crosslinked by EDC/NHS ethanol solution (EDC: 10 mg/mL, NHS: 6 mg/mL) over night. 10 wt% PCL solution was electrospun for 5 min on the surface of Fluorescein-loaded PCL/gelatin nanofiber (∅12 mm) attached onto the rotating collector (needle: 18 G; flow rate: 2 mL/h; needle-to-collector distance: 15 cm; voltage: 15 kV; collector diameter: 75 mm, rotating speed: 40 rpm). Fluorescein-loaded Janus nanofiber was fixed in the diffusion test cell (bottom compartment was filled with 1050 μL of PBS solution). Solution in the bottom compartment was collected at 0.5, 1, 2, 4, 6, 12 and 24 h later, and fluorescence intensity was measured (excitation wavelength: 494 nm, emission wavelength: 521 nm).

#### 2.2.5. Antibacterial Property Test (Colony Count Assay)

A hundred microliter of *E. coli* suspension or *S. aureus* suspension (10^4^ CFU/mL) added on the surface of PCL/gelatin, Janus and Cur Janus nanofiber (∅12 mm) incubated at 37 °C for 24 h. After incubation, the nanofiber was soaked with 1 mL of PBS to collect survived *E. coli* suspension or *S. aureus* suspension. A hundred microliter of collected *E. coli* or *S. aureus* suspension was seeded on the LB culture plate and cultured at 37 °C overnight. The colonies on the LB plate were counted to calculate the concentration of bacterial suspension and antibacterial rate is calculated by the following equation (N*_control_*: colony number when directly seeded on LB plate; N*_n_*: colony number processed on the PCL/gelatin, Janus and Cur Janus nanofiber):

(3) $$\mathrm{Antibacterial}\;\mathrm{rate}\;(\%)\;=\;\frac{N_{control}-N_{n}}{N_{control}}\times 100$$

#### 2.2.6. Antioxidant Property Test (DPPH Radicals Scavenging Ability)

Eighty micromolar DPPH ethanol solution was prepared by dissolving 0.8 mg of DPPH powder in 25 mL of ethanol. Nanofibers (PCL/gelatin, Janus, Cur Janus, ∅12 mm, approximately 3 mg) were soaked into 2 mL of DPPH ethanol solution, respectively, and incubated in the dark for 30 min at room temperature. The absorbance of the solution with or without soaking the fibers into DPPH ethanol solution were measured at 517 nm by UV–vis spectrophotometer. The background absorbance (A*_background_*) of PCL/gelatin, Janus and Cur Janus is determined by soaking nanofibers (PCL/gelatin, Janus, Cur Janus, ∅12 mm, approximately 3 mg) in 2 mL of DPPH-free ethanol for 30 min, respectively. Radical scavenging rate is calculated by the formula below:

(4) $$\mathrm{DPPH}\;\mathrm{radical}\;\mathrm{scavenging}\;\mathrm{rate}\;(\%)\;=\;\left(1-\frac{A_{sample}-A_{background}}{A_{DPPH}-A_{background}}\right)\times 100$$

A: absorbance of the samples.

#### 2.2.7. Biocompatibility Test of Janus Nanofiber

Nanofibers (PCL/gelatin nanofiber, Janus nanofiber and Cur Janus nanofiber, ∅12 mm, 3 mg) were immersed in 5 mL of D-MEM for 24 h at 37 °C (*n* = 3). NIH/3T3 cell were seeded at 8000 cells/well in 96-well plate (0.33 cm^2^/well) and culture for 24 h with 100 μL/well of culture media (10 vol% FBS-containing D-MEM). The culture media was replaced with 100 μL of leach liquor of nanofiber mixed with 10 vol% of fresh FBS and cultured for 48 h. WST-8 assay was performed using CCK-8 kit to evaluate the live cell activity (mitochondrial activity) under manufactured protocol.

#### 2.2.8. Wound Healing Test for Diabetic Model Mouse

ICR mice (male, 6 weeks old, SLC, Shizuoka, Japan) weighing about 28–30 g were used to induce diabetes. The experimental procedures followed protocols sanctioned by the Ethics Committee on Animal Experiments of Kyushu University (A25-302-0).

Mice were housed in a temperature-controlled environment (25 ± 1 °C) with a 12 h light/dark cycle. After 12 h fasting, mice were received intraperitoneal injections with streptozotocin (STZ) at a dose of 80 mg/kg body weight at Day 1 and Day 3. STZ has specific toxic to pancreatic *β* cells and was widely used to establish an animal model of diabetes in medical research [35]. The blood glucose level and weight of each mouse was measured weekly for 4 weeks. When the fasting blood glucose level exceeded 300 mg/dL, the mice were considered to successfully induced diabetes, and then the wound healing test was performed.

A total of twelve mice were used for in vivo full-thickness skin wound healing (four healthy mice, eight diabetic mice). Before the wounds were created, all the mice were anesthetized with exposure to isoflurane and shaved on the back. The mouse excisional wound healing model is used to study wound healing, but mouse skin contraction accounts for a large part of wound closure unlike human beings [36]. To minimize the impact of wound contraction on the regeneration of wound tissue, two silicone rings (inner diameter: 10 mm) were bonded with cyanoacrylate instant glue and fixed with four sutures. Then two full-thickness wounds (∅8 mm) were created with punch and surgical scissors on the back of each mouse. Therefore, the wound heals through granulation and re-epithelialization, a process similar to that occurring in humans. Four mice were used for each group. For the healthy group: *n* = 4, Healthy; healthy mice treated with medical gauze and Tegaderm. The diabetic mice were randomly divided into two groups (*n* = 4, Cur; diabetic mice treated with Cur Janus nanofiber, No treatment; diabetic mice treated with medical gauze and Tegaderm). The wound dressing was replaced on Day 3, Day 6 and Day 12, and the wound area was determined using wound photo analysis. The wound area of each mouse was analyzed by using Fiji-ImageJ software (2.14.0). The animals were sacrificed on Day 12.

For the histological analysis, the wound tissue was fixed in formalin and sliced into 6 μm sections after paraffin embedding. These samples were subjected to hematoxylin and eosin (H&E) staining for skin tissue analysis (*n* = 4). The sections were also stained against CD68 and CD206 for immunohistochemical analysis (*n* = 4).

#### 2.2.9. Statistical Analysis

All the quantified data were presented as the mean ± standard deviation (S.D.). At least three independent experiments were carried out for all experiments. The statistical difference was evaluated by using one-way ANOVA with Tukey’s multiple comparison test.

## 3. Results and Discussion

### 3.1. Janus Nanofiber Preparation and Characterization

**Figure 2:**
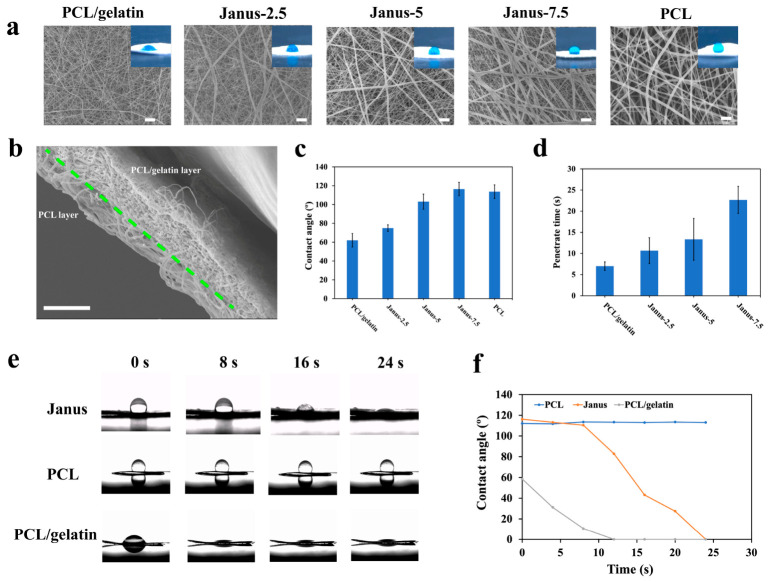
Fundamental properties of Janus nanofiber dressings based on PCL/gelatin and PCL nanofibers. (**a**) SEM images of hydrophilic nanofiber (PCL/gelatin), Janus nanofiber (2.5~7.5 min) and hydrophobic layer (PCL) (scale bars = 10 µm). (**b**) SEM images of Janus nanofiber’s section (scale bars = 50 µm, dashed line: boundary line between PCL and PCL/gelatin layer). (**c**) Water contact angle of hydrophilic nanofiber (PCL/gelatin), Janus nanofiber (PCL side) and PCL nanofiber. (**d**) Water penetration time on hydrophilic nanofiber and Janus nanofiber (from PCL side) (*n* = 3, Bars: S.D.). (**e**) The wetting behaviors of PCL/gelatin nanofiber, PCL nanofiber and hydrophobic surfaces of Janus nanofiber (7.5 min). (**f**) Water contact angle change on PCL, Janus and PCL/gelatin nanofiber with time (*n* = 1).

Figure 2a shows the micro-morphology of the Janus nanofibers composed of PCL fibers and PCL/gelatin fibers. The average fiber diameter of the PCL/gelatin (1:2 *w*/*w*) nanofiber is 0.46 ± 0.07 μm and the average pore size is 0.91± 0.63 μm (Figure S1), while PCL nanofiber’s diameter is 2.19 ± 0.42 μm (Figure S4 and Table S1). The PCL layer was electrospun onto the surface of the PCL/gelatin nanofibers with an electrospinning time of 0, 2.5, 5, and 7.5 min, respectively. With prolonged electrospinning time, the fiber density of the PCL nanofiber increased, and thereby nanofiber pore size reduced (Figure 2a and Figure S4). In addition, the pore size distribution of PCL nanofibers side is mainly concentrated between 10–50 μm (Figure S4c), 57.14% of Janus-2.5, 65.52% of Janus-5 and 67.33% of Janus-7, respectively, whereas the average pore size of the PCL/gelatin nanofiber side is 906 ± 62 nm (Table S1). A clear bilayer structure could also be observed in the sectional image (Figure 2b). With increasing electrospinning time, the static water contact angle (SWCA) at the PCL side of the Janus nanofibers increased from 62.01° to 116.49°. Notably, at electrospinning times of 5 and 7.5 min (Janus-5 and Janus-7.5), the PCL side of the Janus nanofibers exhibited clear hydrophobic characteristics (SWCA > 90°) (Figure 2c). PCL/gelatin nanofibers and PCL nanofibers exhibit completely opposite wettability (Figure S2). Although PCL/gelatin (1:2 *w*/*w*) nanofiber shows lower water uptake value compared with those of the nanofiber with higher gelatin ratio, it maintains porous structure well after hydration and lower mass loss (Figure S3).

To verify the unidirectional fluid transportation ability of Janus nanofiber, methylene blue aqueous solution droplets was added to the PCL side of the Janus nanofiber to observe the fluid transport behavior. As a result, the hydrophobicity of the Janus hydrophobic layer increased, the fluid penetration time became longer, making penetration more difficult. Janus-5 exhibited a faster penetration rate compared with Janus-7.5 and was more effective in achieving unidirectional liquid transportation (Figure 2c,d). The wetting behavior of PCL/gelatin, PCL and Janus nanofibers and water contact angle change with time are shown in Figure 2e,f, the wide range of SWCA changes also proves that fluid could be transported from the hydrophobic layer into the hydrophilic layer. This unique unidirectional penetration endows the Janus dressing with great potential for the treatment of chronic wounds with excessive exudate.

### 3.2. Unidirectional Transportation Behavior Observation

**Figure 3:**
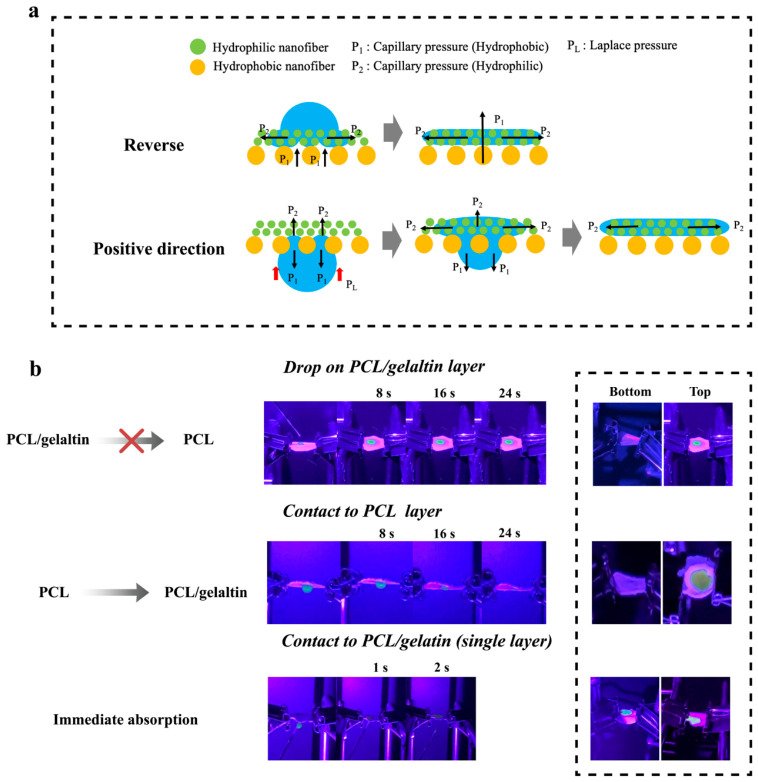
Unidirectional fluid transporting properties of Janus nanofiber. (**a**) Illustration of the unidirectional liquid transport mechanism. (**b**) The fluorescent images of the fluorescent droplet contacting with hydrophilic nanofibers (PCL/gelatin) and Janus nanofiber (PCL/gelatin side and PCL side), respectively.

To mimic the exudate management conditions of diabetic wounds, the unidirectional water transport of the Janus nanofibers was visualized with a 1 wt% fluorescein sodium PBS solution. When the fluorescent droplet was contacted to the hydrophilic side of the Janus nanofiber (PCL/gelatin layer), rapid spreading of fluorescence was observed on the hydrophilic side within 8 s, while no fluorescence was detected on the hydrophobic side, indicating that PBS did not penetrate through the Janus nanofiber due to the hydrophobic layer’s blockage in PCL layer. In contrast, when the fluorescent droplet was brought into contact with the hydrophobic side of the Janus nanofiber (PCL layer) perpendicularly from below, the transport process can be observed in 24 s, and all the fluorescent fluid was transported to the hydrophilic layer. For the single-layer PCL/gelatin nanofiber, when the fluorescent droplet contacted the nanofiber from below, rapid penetration occurred, and fluorescence was observed on both sides, which means there is no transportation but absorption in single hydrophilic layer nanofiber. These results further demonstrate that, in the Janus nanofiber, water transport occurs exclusively from the hydrophobic side toward the hydrophilic side and shows the Janus nanofiber’s superiority to single-layer hydrophilic nanofiber (Figure 3b).

We speculate that this phenomenon is governed by the combined effects of unbalanced capillary forces and Laplace pressure (Figure 3a). According to the Young–Laplace Equation (5),

(5) $$P_{L}=\frac{2\gamma }{r}$$

*γ* is the liquid–gas interfacial tension (water, 0.072 N m^−1^, 25 °C), the upward Laplace pressure acting on a water droplet is inversely proportional to the radius of curvature (r) of the water–air interface [37,38,39].

As show in Figure 3a, when a water droplet initially contacts the hydrophobic side (PCL layer) with a WSCA > 90°, the Laplace pressure drives the droplet into the voids of the PCL nanofiber, forming a hydrophobic–hydrophilic contact point. Once this contact is established, the transport process is triggered, and the capillary force of the hydrophilic nanofibers subsequently draws the liquid inward, allowing the transport to proceed. In contrast, when a droplet is placed on the hydrophilic side (PCL/gelatin layer), water readily spreads within the hydrophilic matrix. However, penetration is hindered upon encountering the PCL nanofiber layer due to the opposing capillary force generated by the hydrophobic fibers. The capillary pressure (P*_c_*) can be calculated with the formula below (6):

(6) $$P_{c}\;=\;\frac{4\gamma }{D}cos\theta$$

where D is the pore size of nanofiber, *γ* is the liquid–gas interfacial tension (water, 0.072 N m^−1^, 25 °C), and *θ* is the contact angle of the liquid droplet on the nanofiber surface [14,20].

Janus-5 has the appropriate pore size of PCL nanofibers (10–50 μm), where the liquid is pushed by the Laplace force on the hydrophobic surface (WSCA > 90°), contacting the hydrophobic–hydrophilic contact points. The hydrophilic capillary force of the small-pore size PCL/gelatin nanofibers (906 ± 62 nm) pulls the liquid, completes the transport, ensures the dryness on the hydrophobic side, and is an ideal candidate for diabetic wound treatment.

Prolonged soaking can lead to overhydration of the surrounding tissue, loss of its protective function, and subsequent infection, thereby delaying wound healing [14,18]. The Janus dressing can prevent excessive exudate from rewetting the peri-wound area, a function that cannot be achieved by conventional hydrophilic wound dressings. We evaluated its ability to drain added 50 μL simulated biofluid (0.05 wt% fluorescein sodium in PBS) in a mouse full thickness wound model and compared it with PCL nanofiber and medical gauze dressing. When the wound was treated with the Janus nanofiber, the simulated biofluid was effectively expelled from the wound bed. Under UV illumination, only a small amount of fluorescent biofluid remained in wound with low fluorescence intensity (Figure S5), indicating that the fluid was pumped into the Janus dressing and could not penetrate the hydrophobic nanofiber array to rewet the wound. In contrast, for the gauze dressing, a larger green fluorescent area formed around the peri-wound, demonstrating that the liquid could easily spread along the dressing and rewet the peri wound tissue, while there was still a large amount exudate remaining in PCL nanofiber treated wound.

### 3.3. Drug Release In Vitro

The poor solubility and low bioavailability of curcumin significantly limit its further application. PCL nanofibers can serve as an effective carrier system for curcumin retention and sustained release. Owing to the low solubility of curcumin in aqueous solutions, its release kinetics were evaluated in a PBS/Tween 80 medium (99.5: 0.5, *v*/*v*). In this case, it is possible to confirm whether all the curcumin incorporated into the PCL nanofiber can be released due to diffusion or adsorption–desorption equilibrium.

**Figure 4:**
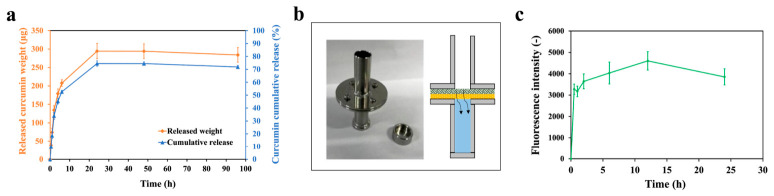
Simulation of double release with hydrophilic and hydrophobic drugs in Janus nanofibers. (**a**) Cumulative curcumin release and released curcumin weight of curcumin from 10 wt% Cur PCL nanofiber in PBS/Tween 80 medium (*n* = 3, Bars: S.D.). (**b**) Photograph and schematic diagram showing the simulation of hydrophilic drug release with diffusion test cell. (**c**) Fluorescence intensity in PBS with the sodium fluorescein release from hydrophilic layer through hydrophobic layer (*n* = 3, Bars: S.D.).

As shown in the Figure 4a, in the initial 6 h, 52.63% (207.73 μg) of curcumin in Cur PCL nanofiber was released, which may attribute to the higher solubility of curcumin on the nanofiber surface leads to larger burst release in a PBS/Tween 80 medium compared to the exudate at skin wounds. This initial burst release was followed by a slower and more sustained release phase, with the cumulative release reaching approximately 74.44% (294.12 μg) after 48 h of incubation, which shows the sustained-release characteristics of Cur PCL nanofiber. However, curcumin release approached a plateau after 48 h, which may be attributed to the limited diffusion of deeply entrapped curcumin in hydrophobic polymer matrix of PCL. The remaining 25.56% of curcumin might potentially be released during long-term matrix degradation, PCL degradation occurs over a much longer timescale than the dressing replacement interval used in this study. These results demonstrate that Cur PCL nanofibers are capable of providing sustain-release after 6 h and sustained release for at least 48 h, indicating the potential drug releasing efficacy of Cur PCL nanofiber in diabetic wound treatment [40,41,42].

To verify the reverse-release behavior of hydrophilic drugs through PCL nanofiber layer from the hydrophilic side of the Janus dressing, fluorescence sodium was incorporated into PCL/gelatin hydrophilic layer. In vitro drug release from PCL/gelatin nanofiber was performed to detect the fluorescence intensity in diffusion test cell (Figure 4b). The fluorescence intensity in the receiving side was quantified within 24 h. As shown in Figure 4c, the fluorescence sodium inside PCL/gelatin was rapidly released through the hydrophobic layer of PCL nanofibers within 1 h, accompanied by a slow release over 23 h. This demonstrates the possibility of releasing hydrophilic small molecule drugs in Janus nanofibers’ hydrophilic layer.

These findings indicate that small-molecule hydrophilic drugs incorporated into the PCL/gelatin hydrophilic layer are able to permeate through the hydrophobic PCL layer and undergo reverse release toward the wound-facing side. Consequently, the Janus dressing is capable of enabling the simultaneous delivery of both hydrophilic and hydrophobic drugs. Hydrophobic drug incorporated Janus dressings were reported recently [43,44,45], hydrophobic drugs endows nanofiber with functionalities and realize acceleration of wound healing. In this study, not only hydrophobic drugs, but also hydrophilic drugs were incorporated in Janus nanofibers, which might enable independent yet coordinated release behaviors, providing sustained tissue-regenerative activity for enhanced diabetic wound healing. Hydrophilic drugs such as dimethyloxalylglycine (175.14 Da), metformin (165.62 Da) and hyaluronan oligosaccharide (under 2000 Da) have been reported for accelerating diabetic wound healing by improving neo-vascularization, re-epithelialization and fibroblast proliferation [46]. Our Janus nanofibers potentially utilize these promising hydrophilic drugs for more advanced wound healing.

### 3.4. Antibacterial and Antioxidant Property of Multifunctional Janus Nanofiber

**Figure 5:**
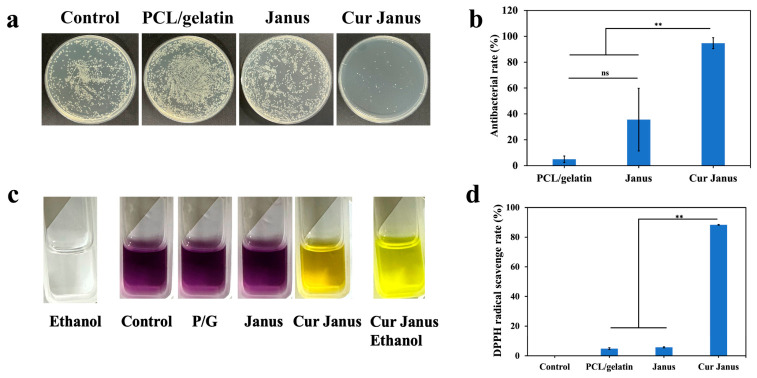
In vitro functional evaluation of Cur Janus nanofiber. (**a**) Photograph of *E. coli* colonies and (**b**) the related antibacterial rate (*n* = 3, Bars: S.D.) for the PCL/gelatin, Janus and Cur Janus nanofibers. (**c**) Images of DPPH scavenging activity using different nanofibers (P/G refers to PCL/gelatin). (**d**) DPPH radicals scavenge rate (*n* = 3, Bars: S.D.) of different nanofibers co-incubated with DPPH ethanol for 30 min. (** *p* < 0.01, ns: not significant).

Antibacterial dressings are considered beneficial for improving the healing outcome of diabetic wounds. Escherichia coli (*E. coli*, Gram-negative) and Staphylococcus aureus (*S. aureus*, Gram-positive) was selected as a model bacterium to evaluate the antibacterial activity of different dressings, including PCL/gelatin, Janus, and Cur Janus, using the serial dilution method and plate counting assay. As shown in Figure 5a and Figure S6, the *E. coli* antibacterial rates of PCL/gelatin and Janus dressings were approximately 4.89% and 35.61%, respectively, whereas Cur Janus exhibited a markedly higher growth inhibition rate of about 94.68% (*p* < 0.01). Similarly, Cur Janus showed highest *S. aureus* antibacterial rate of 96.98%, which significantly higher than PCL/gelatin and Janus nanofiber group (*p* < 0.01). This may attribute to the sustain release of curcumin from Curcumin/PCL layer of Cur Janus nanofiber, which maintains an effective local concentration of curcumin at the bacteria-nanofiber interface. Curcumin could disrupt bacterial cell membrane and lead to leakage of membrane in Gram-negative and Gram-positive bacteria, which effectively inhibit the growth of bacteria on Cur Janus nanofiber [29]. These results indicate that the Cur Janus dressing possesses a pronounced antibacterial effect against *E. coli* and *S. aureus*, suggesting its potential to suppress bacterial infection during the wound healing process.

Oxidative stress, primarily induced by prolonged inflammation and bacterial infection, represents a major impediment to diabetic wound healing. As shown in Figure 5b, no noticeable color change of the deep purple DPPH solution was observed in the presence of Janus or PCL/gelatin dressings. In contrast, the purple color of the DPPH solution was markedly diminished when incubated with the curcumin-containing Cur Janus dressing. The absorbance at 517 nm was measured after incubation, and the DPPH radical scavenging efficiency was calculated. Approximately 88.32% of DPPH radicals were scavenged by Cur Janus, demonstrating its pronounced free radical scavenging capability (Figure 5d). These results indicate that Cur Janus is effective in mitigating oxidative stress in vitro, suggesting its potential relevance for creating a favorable microenvironment during the diabetic wound healing process.

### 3.5. Biocompatibility of Janus Nanofiber

**Figure 6:**
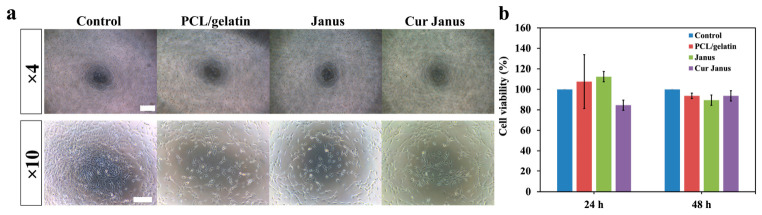
Biocompatibility of different nanofibers. (**a**) Cell morphology of 3T3 cells incubated with the PCL/gelatin, Janus and Cur Janus extracts for 24 h (*n* = 4); scale bars = 400 μm, scale bars in enlarge images (×10) of 24 h; scale bars = 200 μm. (**b**) Cell viability of 3T3 cells incubated with the PCL/gelatin, Janus and Cur Janus extracts at different time point (*n* = 4, Bars: S.D.).

Excellent biocompatibility is a fundamental prerequisite for the application of Janus dressings in wound management. As shown in Figure 6b, the WST-8 assay revealed that the viability of NIH/3T3 fibroblasts remained above 80% after incubation for 24 and 48 h with extracts of PCL/gelatin, Janus, and Cur Janus dressings at a concentration of 0.6 mg/mL. In addition, typical fibroblast morphology was clearly observed under optical microscopy. These results indicate that the Cur Janus dressing exhibits good cytocompatibility, supporting its suitability for wound healing-related applications.

### 3.6. Diabetic Wound Healing Analysis

We further investigated whether the multifunctional Cur Janus dressing could enhance diabetic wound healing in vivo. An in vivo diabetic wound model was established by intraperitoneal injection of STZ into mice. When blood glucose levels persistently exceeded 300 mg/dL, full-thickness excisional wounds (diameter = 8 mm) were created on the dorsal skin of the mice and stabilized with splints to minimize the influence of skin contraction on wound closure.

**Figure 7:**
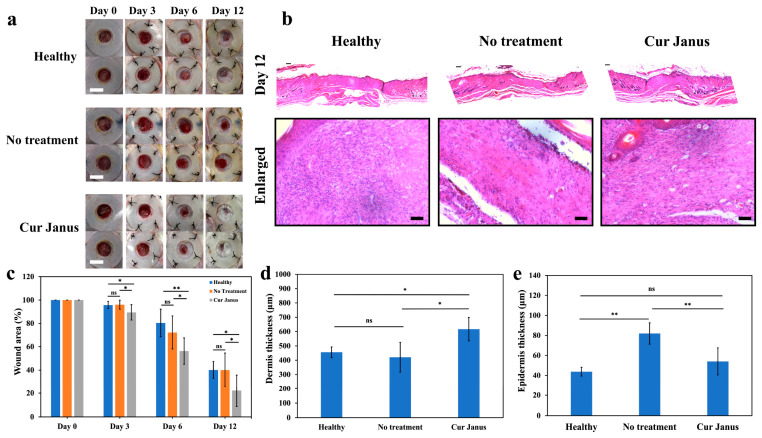
Accelerated wound healing process. (**a**) Representative images of wound healing in healthy mice (Gauze treated), diabetic mice (Gauze treated) and diabetic mice (Cur Janus treated) groups; scale bars = 8 mm. (**b**) H&E stained sections of regenerated tissue in healthy mice (Gauze treated), diabetic mice (Gauze treated) and diabetic mice (Cur Janus treated) groups on Day 12; scale bars = 200 μm (the enlarged images in the box area; scale bars = 50 μm). (**c**) Wound area was measured at Day 0, Day 3, Day 6 and Day 12. (*n* = 4, Bars: S.D.). (**d**,**e**) Quantification of the dermis and epidermis thickness of regenerated tissue on Day 12 (*n* = 4, Bars: S.D.). All statistical analyses were performed using one-way ANOVA followed by Tukey’s multiple comparison test, * *p* < 0.05, ** *p* < 0.01, ns: not significant.

Blood glucose levels were continuously monitored throughout the healing process, and diabetic mice in both the No treatment and Cur Janus group maintained glucose levels above 300 mg/dL during the experimental period. Wound healing performance was subsequently evaluated in healthy mice (Healthy), diabetic mice without treatment (gauze + Tegaderm, No treatment), and diabetic mice treated with Cur Janus. As shown by representative wound images and quantitative wound area analysis in Figure 7a,c, the Cur Janus–treated group exhibited a significantly smaller wound area compared with the No treatment and gauze groups, indicating accelerated wound closure in diabetic mice. On Day 6, the wound area in the Cur Janus group decreased by approximately 56.30%, whereas only about 20% reduction was observed in the No treatment and gauze groups. By Day 12, wounds treated with Cur Janus were largely regenerated, with a residual wound area of approximately 22.20%, while the No treatment group still exhibited about 40% unhealed wound area.

The tissue regeneration during the wound healing process was analyzed using histopathological staining techniques. As shown in the H&E-stained tissue sections in Figure 7b, the Cur Janus group exhibited more mature dermal tissue and a higher number of fibroblasts compared to the No treatment group on Day 12 of wound healing. As shown in Figure 7d,e, the Cur Janus group showed a thicker dermal thickness than the No treatment group, indicating an enhanced degree of tissue repair in Cur Janus group. In contrast, the No treatment group displayed a thicker epidermal layer, suggesting persistent epidermal hyperplasia and reflecting delayed wound healing [18].

One limitation of this study is the relatively small sample size (*n* = 4 per group) used in the in vivo experiments. Although significant differences were observed among the groups, studies with larger animal cohorts are needed to further strengthen the statistical robustness and validate the therapeutic efficacy of the proposed dressing.

**Figure 8:**
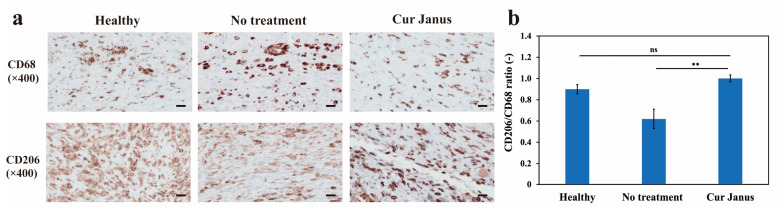
Immunohistochemical staining of wounds in the Healthy, No treatment and Cur Janus groups. (**a**) The immunohistochemical images of regenerated tissues stained with CD68 (total macrophages) and CD206 (M2 macrophage) at Day 12 (scale bars = 20 μm). (**b**) The CD206/CD68 ratio in the Healthy, No treatment and Cur Janus groups. (*n* = 4, Bars: S.D.) The results in histograms are plotted as the mean± S.D. (** *p* < 0.01, ns: not significant).

In the wound healing process of diabetic wound, the transition from proinflammatory (M1) to proregenerative (M2) macrophage phenotype is usually delayed. Therefore, promoting M2 macrophage polarization is a key factor in enhancing diabetic wound healing [47]. Curcumin has been demonstrated to effectively regulate macrophage polarization in vivo by increasing the expression of M2 macrophages while reducing the expression of M1 macrophages, thereby suppressing inflammation [48]. As shown in Figure 8a, wound tissue sections were immunostained for CD68 and CD206. CD206 is normally expressed on the M2 macrophages, while CD68 is expressed on all types of macro-phages, therefore ratio of CD206 and CD68 positive cell numbers could reflect tissue regeneration stage in wound (Figure 8b). Cur Janus group shows higher M2 macrophage ratio than No treatment group (*p* < 0.01) and shows no significance with healthy group. This indicates that the synergistic effects of curcumin release and exudate drainage from the Cur Janus nanofibers can effectively suppress inflammation in diabetic wounds and accelerate wound healing.

## 4. Conclusions

In this study, we developed a Janus nanofibrous wound dressing for diabetic wound healing, featuring exudate management capability, dual-release potential for hydrophilic and hydrophobic agents, as well as anti-inflammatory, antibacterial, and antioxidant properties. Its unidirectional fluid transport behavior was demonstrated both in vitro and in vivo, effectively preventing excessive wound exudate maceration. Curcumin was incorporated into the hydrophobic PCL layer and in vitro release studies exhibited its sustained release. Meanwhile, the presence of curcumin endowed the Janus nanofibrous dressing with excellent antibacterial and antioxidant activities.

To assess the therapeutic efficacy of Cur Janus, it was applied to a full-thickness skin wound model in diabetic mice. The results showed that Cur Janus not only accelerated wound closure but also effectively suppress inflammation in diabetic wound. Finally, the capability of this Janus nanofiber system to simultaneously deliver both hydrophilic and hydrophobic bioactive agents was validated, providing a foundation for further development based on this platform.

Overall, the Cur Janus nanofibrous dressing, as a new-generation wound dressing, exhibits promising potential for chronic wound healing.

## Data Availability

Data are contained within the article and Supplementary Materials.

## Supplementary Materials

The following supporting information can be downloaded at: https://www.mdpi.com/article/10.3390/biom16060902/s1, Figure S1: Micro-morphologies of PCL/gelatin nanofiber. (a) SEM images of PCL/gelatin nanofiber (scale bars = 10 μm). (b) Diameter distribution of PCL/gelatin nanofiber. (c) Pore size distribution of PCL/gelatin nanofiber; Table S1: Diameter and pore size of PCL/gelatin nanofiber with different PCL/gelatin ratio; Figure S2: Water contact angle test of PCL/gelatin nanofiber and contact angle change with time. (a,b) Photo of contact angle change on the surface of PCL/gelatin nanofiber in 9 s. (c) Initial contact angle upon first contact with PCL/gelatin nanofiber (*n* = 3, Bars: S.D.); Figure S3: Water uptake and degradation of PCL/gelatin nanofiber. (a) SEM images of crosslinked PCL/gelatin nanofiber (Freeze dried and water hydrate) (scale bars = 10 μm). (b) Water uptake value of PCL/gelatin nanofiber with different PCL/gelatin ratio (*n* = 3, Bars: S.D.). (c) Degradation of PCL/gelatin nanofiber in water (*n* = 3, Bars: S.D.); Figure S4: Fiber diameter and pore size distribution of Janus nanofiber. (a) Diameter distribution of PCL nanofiber. (b) SEM images of Janus nanofiber with different PCL nanofiber electrospinning time (2.5, 5 and 7.5 min). (c) Pore size distribution of PCL nanofiber with different electrospinning time (2.5, 5 and 7.5 min) (scale bars = 10 μm); Figure S5: Unidirectional transporting properties in vivo. (a) The picture of wound tissue with fluorescent-labeled simulated exudate treated with Janus nanofiber, PCL nanofiber and medical gauze. (b) Mean fluorescence intensity in wound area of control, PCL, gauze and Janus group (*n* = 3, Bars: S.D.); Figure S6. Antibacterial ability of Cur Janus nanofiber to Gram-positive bacteria (*S. aureus*). (a) Photograph of *S. aureus* colonies and (b) the related antibacterial rate (*n* = 3, Bars: S.D.) for the PCL/gelatin, Janus and Cur Janus nanofibers; Figure S7. Water vapor transmission rate (WVTR) of PCL, PCL/gelatin and Cur Janus nanofibers at 32 °C (*n* = 3, Bars: S.D.); Figure S8. Evaluation of mechanical properties of Cur Janus nanofiber in dry and hydrated state. (a) Typical tensile stress and strain curves of Cur Janus nanofiber. (b) Tensile strength of Cur Janus nanofiber (*n* = 3, Bars: S.D.); Table S2. Mechanical properties of Cur Janus nanofiber in dry and hydrated state (*n* = 3, Bars: S.D.) [49,50,51,52].

## Abbreviations

The following abbreviations are used in this manuscript:

Cur	Curcumin
PCL	Poly(ε-caprolactone)
ROS	Reactive oxygen species
HFIP	Hexafluoro isopropanol
EDC	1-Ethyl-3-(3-dimethylaminopropyl) carbodiimide
NHS	N-Hydroxy succinimide
DMF	N, N-Dimethylformamide
PBS	Phosphate buffered saline
DPPH	2,2-diphenyl-1-picrylhydrazyl
FBS	Fetal bovine serum
H&E	Hematoxylin and eosin
SWCA	Static water contact angle
STZ	Streptozotocin
