# Supplementary material for: Multifunctional Self-Pumping Janus Dressing for Exudate Management and Diabetic Wound Healing
Source: Biomolecules. 2026 Jun 18;16(6):902. doi: 10.3390/biom16060902 (PMC13297552; doi:10.3390/biom16060902)
Supplement: Supplementary file 1 [file biomolecules-16-00902-s001.zip › biomolecules-4343218-supplementary.pdf]

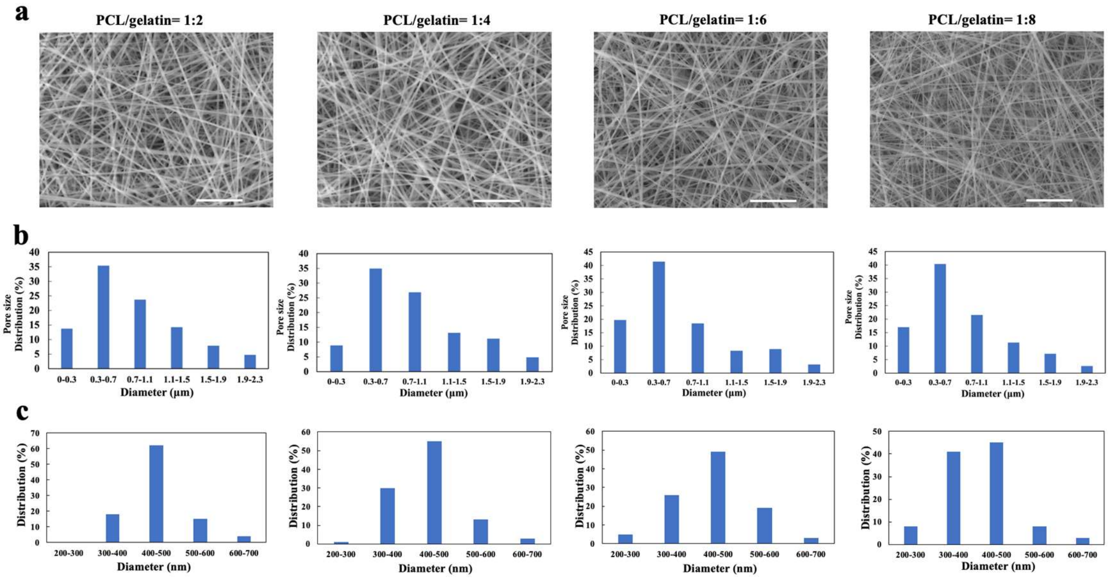

Figure S1. Micro-morphologies of PCL/gelatin nanofiber. a) SEM images of PCL/gelatin nanofiber (scale bars= 10 μm). b) Diameter distribution of PCL/gelatin nanofiber. c) Pore size distribution of PCL/gelatin nanofiber.

Table S1. Diameter and pore size of PCL/gelatin nanofiber with different PCL/gelatin ratio.

PCL/gelatin ratio	Mean diameter (nm)	Mean pore size (*nm*)
1:2	457±67	906±62
1:4	438±72	981±66
1:6	438±80	802±65
1:8	412±81	850±70

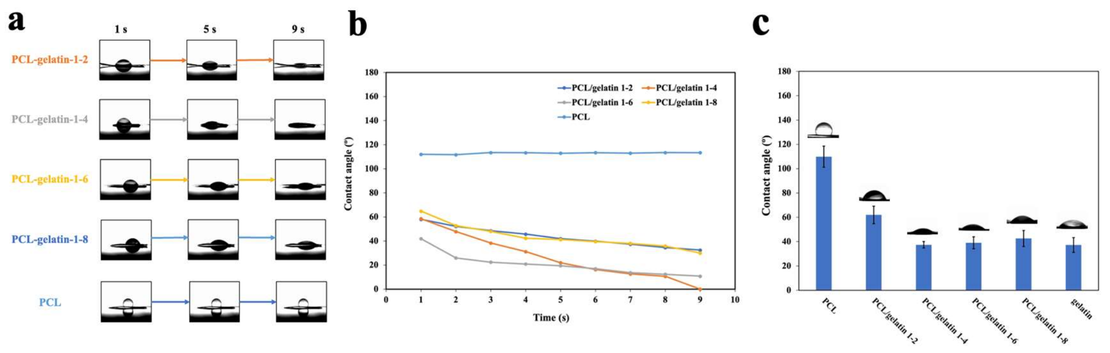

Figure S2. Water contact angle test of PCL/gelatin nanofiber and contact angle change with time. a, b) Photo of contact angle change on the surface of PCL/gelatin nanofiber in 9 s. c) Initial contact angle upon first contact with PCL/gelatin nanofiber (*n*=3, Bars: S.D.).

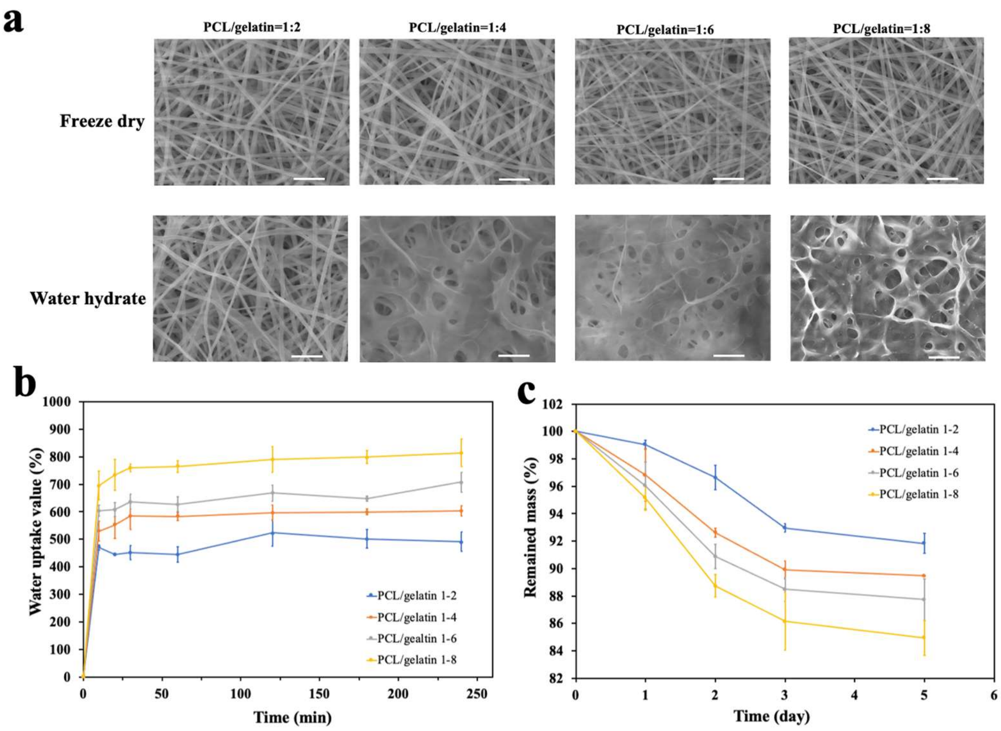

Figure S3. Water uptake and degradation of PCL/gelatin nanofiber. a) SEM images of crosslinked PCL/gelatin nanofiber (Freeze dried and water hydrate) (scale bars= 10 μm). b) Water uptake value of PCL/gelatin nanofiber with different PCL/gelatin ratio (*n*=3, Bars: S.D.). c) Degradation of PCL/gelatin nanofiber in water (*n*=3, Bars: S.D.).

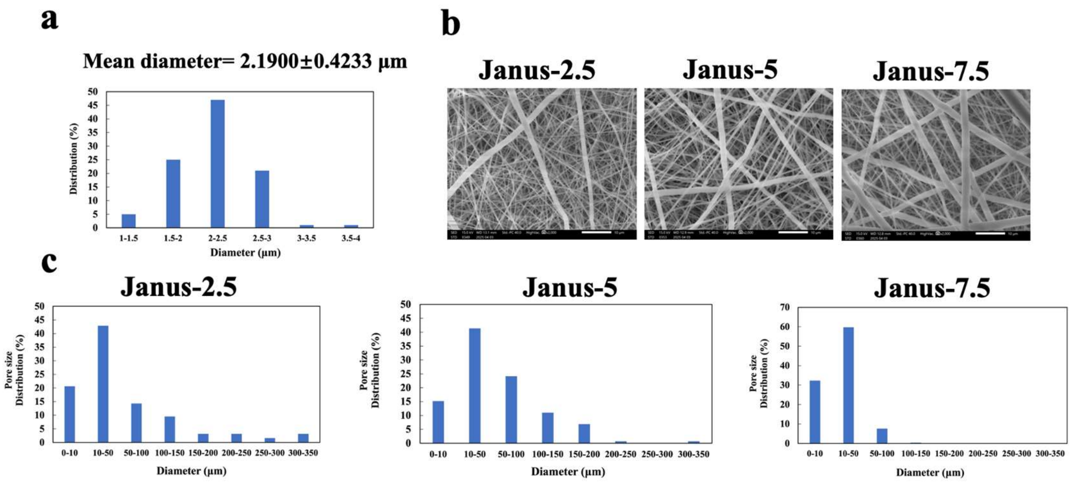

Figure S4. Fiber diameter and pore size distribution of Janus nanofiber. a) Diameter distribution of PCL nanofiber. b) SEM images of Janus nanofiber with different PCL nanofiber electrospinning time (2.5, 5 and 7.5 min) (scale bars= 10 μm). c) Pore size distribution of PCL nanofiber with different electrospinning time (2.5, 5 and 7.5 min).

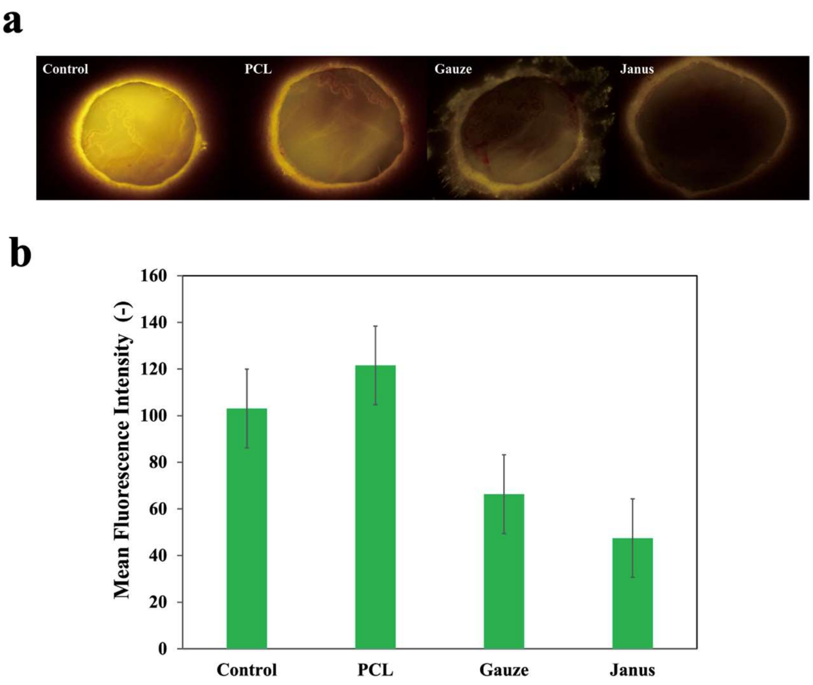

Figure S5. Unidirectional transporting properties *in vivo*. a) The picture of wound tissue with florescent-labeled simulated exudate treated with Janus nanofiber, PCL nanofiber and medical gauze. b) Mean fluorescence intensity in wound area of control, PCL, gauze and Janus group (*n*=3, Bars: S.D.).

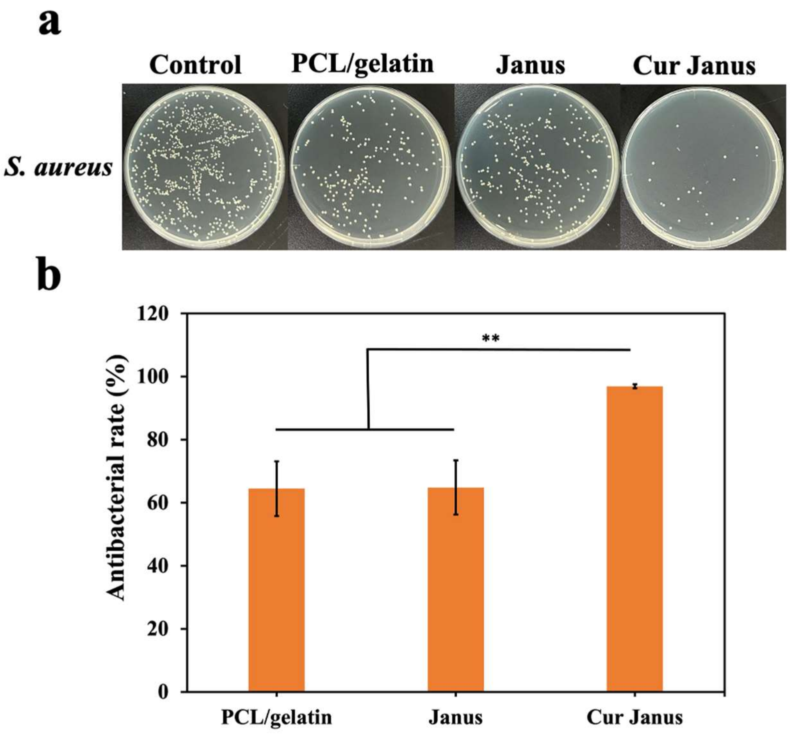

Figure S6. Antibacterial ability of Cur Janus nanofiber to Gram-positive bacteria (*S. aureus*). a) Photograph of *S. aureus* colonies and b) the related antibacterial rate (*n*=3, Bars: S.D.) for the PCL/gelatin, Janus and Cur Janus nanofibers, ** $p < 0.01$.

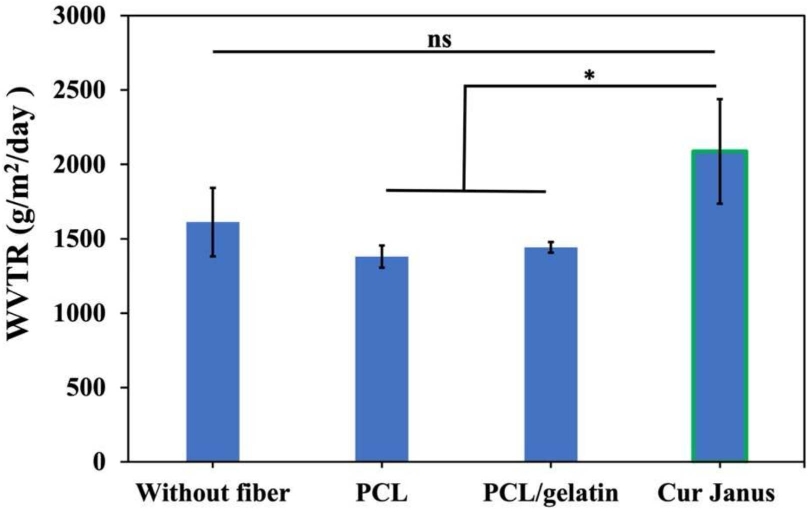

Figure S7. Water vapor transmission rate (WVTR) of PCL, PCL/gelatin and Cur Janus nanofibers at 32 °C (n=3, Bars: S.D.), * $p < 0.05$, ns: not significant.

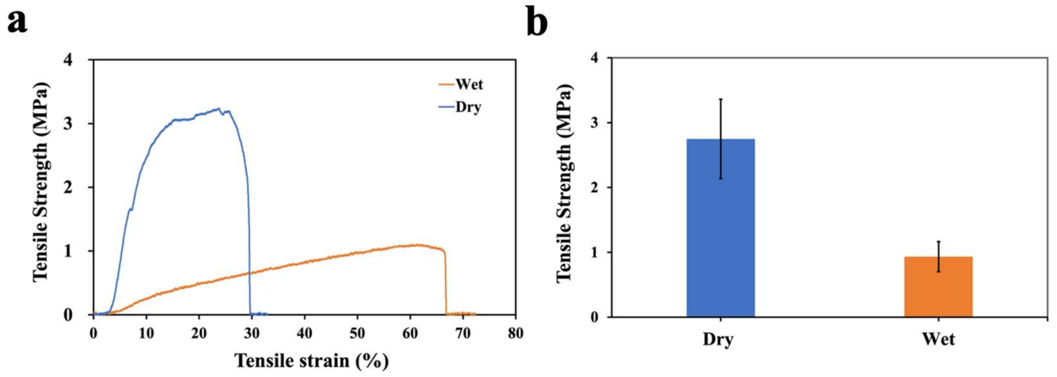

Figure S8. Evaluation of mechanical properties of Cur Janus nanofiber in dry and hydrated state. a) Typical tensile stress and strain curves of Cur Janus nanofiber. b) Tensile strength of Cur Janus nanofiber (n=3, Bars: S.D.).

Table S2. Mechanical properties of Cur Janus nanofiber in dry and hydrated state (n=3, Bars: S.D.).

Conditions	Tensile strength (MPa)	Young's modulus (MPa)	Elongation (%)
Dry	2.75±0.61	32.10±1.40	24.38±5.94
Wet	0.93±0.23	2.80±1.08	55.73±10.31

Supplementary discussion

Water Vapor Transmission ability of Cur Janus nanofiber. The data in Figure S7 indicates that the PCL, PCL/gelatin and Cur Janus nanofiber have WVTR of 1380.47± 74.04, 1442.00± 35.49 and 2086.75± 352.17 g/m²/day, respectively. Such WVTR basically meets the requirement of wound dressing to prevent excessive dehydration and the accumulation of exudates in the wound, which is 2000–2500 g/m²/day [49,50]. Based on this result, Cur Janus nanofiber is expected to be used as diabetic wound dressing with unidirectional exudate draining ability.

Mechanical properties of Cur Janus nanofiber. The tensile strength, Young's modulus and elongation have been investigated (Figure S8, Table S2). In the dry state, the Cur Janus nanofibers exhibited higher tensile strength (2.75± 0.61 MPa) and Young's modulus (32.10± 1.40 MPa), but a lower elongation (24.38± 5.94%). In the hydrated state, water molecules increase the distance between polymer chains in PCL/gelatin nanofiber and reduce the resistance to chain segment mobility, thereby softening the rigid structure of the nanofibers [51]. Macroscopically, this is manifested as a decrease in Tensile strength (0.93± 0.23 MPa) Young's modulus (2.80± 1.08 MPa) and a potential increase in elongation (55.73± 10.31%), as show in Figure S8a. As a wound dressing, appropriate mechanical properties provide the necessary physical integrity to support and promote wound healing. An ideal wound dressing should possess a tensile strength of 2.5–16 MPa, an elongation at break of about 70%, and a Young's modulus within the range of human skin (4.6–20 MPa), allowing it to accommodate skin deformation and support the wound-healing process [52]. The mechanical performance of the Cur Janus dressing was within the approximately desirable range for wound dressing applications, suggesting that it is a suitable candidate for diabetic wound management and wound-healing promotion.

References

49. Pyun, D.G.; Choi, H.J.; Yoon, H.S.; Thambi, T.; Lee, D.S. Polyurethane Foam Containing rhEGF as a Dressing Material for Healing Diabetic Wounds: Synthesis, Characterization, in Vitro and in Vivo Studies. *Colloids Surf. B: Biointerface* **2015**, *135*, 699–706. https://doi.org/10.1016/j.colsurfb.2015.08.029.
50. Xu, R.; Xia, H.; He, W.; Li, Z.; Zhao, J.; Liu, B.; Wang, Y.; Lei, Q.; Kong, Y.; Bai, Y.; et al. Controlled Water Vapor Transmission Rate Promotes Wound-Healing via Wound Re-Epithelialization and Contraction Enhancement. *Sci. Rep.* **2016**, *6*, 24596. https://doi.org/10.1038/srep24596.
51. Gong, X.; Ding, M.; Gao, P.; Liu, X.; Yu, J.; Zhang, S.; Ding, B. High-Performance Liquid-Repellent and Thermal–Wet Comfortable Membranes Using Triboelectric Nanostructured Nanofiber/Meshes. *Adv. Mater.* **2023**, *35*, 2305606. https://doi.org/10.1002/adma.202305606.
52. Ahmad, N. In Vitro and In Vivo Characterization Methods for Evaluation of Modern Wound Dressings. *Pharmaceutics* **2022**, *15*, 42. https://doi.org/10.3390/pharmaceutics15010042.
